# Median-Based Absolute Quantification of Proteins Using Fully Unlabeled Generic Internal Standard (FUGIS)

**DOI:** 10.1021/acs.jproteome.1c00596

**Published:** 2021-11-22

**Authors:** Bharath Kumar Raghuraman, Aliona Bogdanova, HongKee Moon, Ignacy Rzagalinski, Eric R. Geertsma, Lena Hersemann, Andrej Shevchenko

**Affiliations:** Max Planck Institute of Molecular Cell Biology and Genetics, Pfotenhauerstrasse 108, 01307 Dresden, Germany

**Keywords:** absolute quantification of proteins, MS Western workflow, proteome-wide quantification

## Abstract

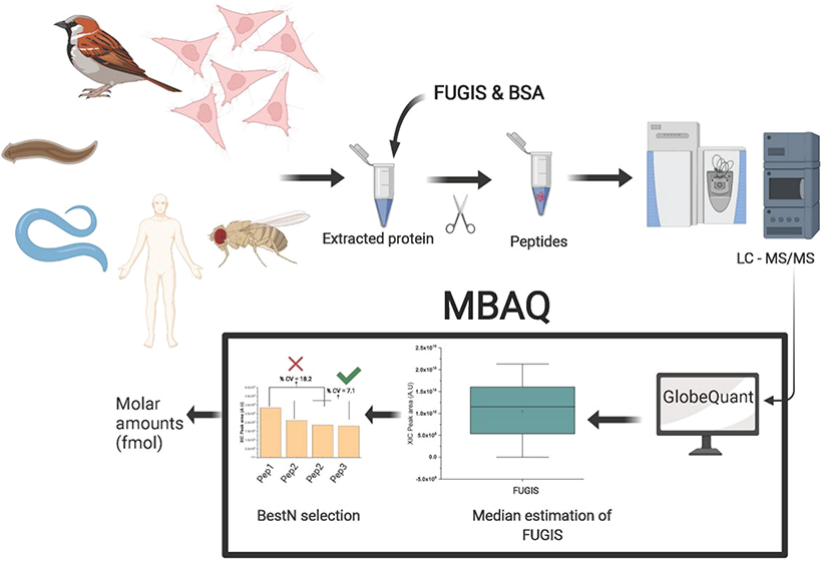

By reporting the molar abundance of proteins, absolute quantification determines their stoichiometry in complexes, pathways, or networks. Typically, absolute quantification relies either on protein-specific isotopically labeled peptide standards or on a semiempirical calibration against the average abundance of peptides chosen from arbitrarily selected proteins. In contrast, a generic protein standard FUGIS (fully unlabeled generic internal standard) requires no isotopic labeling, chemical synthesis, or external calibration and is applicable to quantifying proteins of any organismal origin. The median intensity of the peptide peaks produced by the tryptic digestion of FUGIS is used as a single-point calibrant to determine the molar abundance of any codigested protein. Powered by FUGIS, median-based absolute quantification (MBAQ) outperformed other methods of untargeted proteome-wide absolute quantification.

## Introduction

Proteomics envelopes multiple workflows for relative and absolute quantification of individual proteins. Relative quantification determines how the abundance of the same protein changes across multiple conditions on a proteome-wide scale. In contrast, absolute quantification determines the exact molar quantity of each protein in each condition. In this way, it is possible to relate the molar abundance of different proteins, estimate their expression level, or determine their stoichiometry within a variety of molecular constellations from stable complexes to organelles or metabolic pathways and interaction networks.^1−10^ Absolute quantification holds an important promise to deliver reference values of individual proteins in liquid and solid biopsies, which is a prerequisite for robust molecular diagnostics.

A broad repertoire of absolute quantification techniques tailored toward common analytical platforms, biological contexts, and research aims was developed.^11−13^ It is usually presumed that the average abundance of a few selected peptides faithfully represents the abundance of the corresponding source protein. In turn, peptides quantification relies either on isotopically labeled standards having exactly the same sequence or on a semiempirical calibration against the abundance of selected (or, alternatively, of all detectable) peptides originating from arbitrarily chosen standard proteins.^12,14^ Targeted approaches are more accurate, yet they only cover a small selection of proteins that cannot be changed during the experiment. The latter methods work proteome-wide; however, they rely on arbitrary assumptions, and their accuracy is biased by experimental conditions and the properties of individual proteins.

AQUA^15^ uses a set of isotopically labeled synthetic peptide standards identical with proteotypic peptides from endogenous proteins. Alternatively, QconCAT,^16^ PSAQ,^17^ PrEST,^18^ PCS,^19^ MEERCAT,^20^ DOSCAT,^21^ and GeLC-based MS Western^22^ employ metabolically labeled protein chimeras that, upon proteolytic cleavage, produce the desired peptide standards. MS Western relies on quantifying multiple proteotypic peptides per protein and validates the concordance of protein determinations by monitoring the intensity ratios between the XIC peaks of the standards and the corresponding endogenous peptides. Common discrepancies in these ratios point to an unreliable quantification and are typically due to miscleaved peptides or unexpected post-translational modifications.

To circumvent isotopic labeling, MIPA^23^ and SCAR^24^ standards use minimal sequence permutation or scrambling. It is assumed that scrambled and endogenous peptides share key physicochemical properties that result in equal instrument response,^25,26^ which depends on the analytical conditions and requires extensive validation.

Advances in robust and reproducible LC-MS/MS have led to the notion that generic measures of a protein’s molar abundance could be deduced either from raw intensities or spectral counts of peptide peaks, e.g., emPAI,^27^ APEX,^28^ SCAMPI.^29,30^ Methods like Top3/Hi-3,^6^ iBAQ,^31^ Proteomic Ruler,^32^ xTop^33^ and Pseudo-IS^34^ use averaged XIC intensities of selected or of all peptides matching the protein of interest. Because of limited interlaboratory consistency, they are mostly used for supporting conventional proteomics workflows.

Hence, there is a need to develop a technology combining the accuracy and precision of the internal standards-based targeted quantification with broad (potentially, proteome-wide) coverage and ease of use of untargeted methods. To this end, we developed an untargeted proteome-wide quantification workflow termed median-based absolute quantification (MBAQ) that rely upon a fully unlabeled generic internal standard (FUGIS) based on the common physicochemical properties of proteotypic peptides.

## Materials and Methods

### Protein Extraction from HeLa Cells

HeLa Kyoto cells were cultured in Dulbecco’s modified Eagle’s medium supplemented with 10% fetal calf serum and 1% penicillin–streptomycin (Gibco Life Technologies). HeLa cells were trypsinized, counted, and washed 2 times with PBS before 1 × 10^6^ cells were lysed for 30 min on ice in either 1 or 0.5 mL of RIPA buffer containing CLAAP protease inhibitors cocktail (10 μg/mL aprotinin, 10 μg/mL leupeptin, 10 μg/mL pepstatin, 10 μg/mL antipain, and 0.4 mM phenylmethylsulfonyl fluoride (PMSF)). Subsequently, the cells were further lysed by passing them 10 times through a 25 Gauge syringe. A postnuclear supernatant was obtained by 15 min centrifugation at 4°C and 14 000*g*. The supernatant was used for further analysis by GeLC-MS/MS (Supporting Information, GeLC-MS) with MS-Western and FUGIS standards in separate experiments.

### Absolute Quantification of HeLa Proteins Using MS Western

Absolute protein quantification was performed using the MS Western protocol.^22^ The total protein content from HeLa cells from both dilutions was loaded onto precast 4–20% gradient 1 mm thick polyacrylamide minigels purchased from Anamed Elektrophorese (Rodau, Germany) for 1D SDS PAGE. Separate gels were run for 1 pmol of BSA and isotopically labeled lysine (K) and arginine (R) incorporated chimeric standard containing 3–5 unique quantitypic peptides from the target proteins The sample was cut into 3 gel fractions, and each fraction was codigested with a known amount of BSA and the chimeric standard using Trypsin Gold, mass spectrometry grade (Promega, Madison). The digest was analyzed using the GeLC-MS/MS workflow (Supporting Information, GeLC-MS/MS). Peptides matching and chromatographic peaks alignment was carried out as described in the Supporting Information (Database search and data processing). The quantification was performed using the software developed in house.^9^

### Absolute Quantification of HeLa Proteins Using MBAQ and FUGIS

Similar to the MS Western experiments, the total HeLa cell lysate from both dilutions was separated by1D SDS PAGE. Separate gels were run for 1 pmol of BSA and the fully unlabeled generic internal standard (FUGIS). The gel lane was cut into three gel slices, and each slice was codigested with a known amount of BSA and FUGIS and analyzed by LC-MS/MS (Supporting Information, GeLC-MS/MS). The on-column amount of FUGIS was 200–400 fmol; the loaded amount of chimeric proteins CP01 and CP02 (Supporting Information, Expression and metabolic labeling of protein standards) was 300 fmol. Peptides matching and chromatographic peaks alignment were carried out as described in the Supporting Information (Database search and data processing). The output .csv files with sequences of matched peptides and areas of their XIC peaks were further processed by GlobeQuant software.

### GlobeQuant Software for MBAQ Quantification

GlobeQuant software was developed as a stand-alone Java script-based application using an in-memory SQL database (https://github.com/agershun/alasql) for fast access and search in the CSV file. GlobeQuant runs on a Windows 7 workstation with 16 GB of RAM and a 4-core processor. The .csv output from the Progenesis LC-MS v.4.1 (Nonlinear Dynamics, UK) with peptide ID’s and their respective raw XIC peak areas was used by GlobeQuant software. A list of FUGIS peptides was provided as an input. The software calculated the molar amount of the FUGIS standard using the scrambled and native peptide pairs of BSA , related it to the median area of XIC peaks of FUGIS peptides. The calculated molar amount of the FUGIS standard was related to the median and further used it as a single-point calibrant.

For BestN quantification, peptides were chosen from a pool of Top3 peptides by calculating the coefficient of variation of all possible combination of Best2 and Best3 by default. If a protein did not contain Top3 peptides, the Top2 peptides were taken as BestN peptides. Proteins identified with one peptide were excluded from the quantification. The BestN combination with the lowest coefficient of variation (<20%) was taken and averaged to provide the molar amounts of the protein. The software package is available at https://github.com/bharathkumar91/GlobeQuant.

## Results and Discussion

### MBAQ Workflow for Absolute Quantification

The MBAQ (median-based absolute quantification) workflow relies on a recombinant protein standard consisting of concatenated peptides whose sequences emulate the physicochemical properties shared by typical proteotypic peptides. Its tryptic cleavage produces peptides in exactly equimolar concentrations,^16,21,35,36^ as evidenced by the time course and relative abundance of the rendered peptides.^22^ Therefore, the peptide concentration could be inferred from the known molar abundance of the chimeric protein.

We therefore propose to determine the median value of the areas of the XIC peaks of the peptides produced from chimeric protein and then use it as a single-point calibrant to calculate the molar abundance of other peptides from any codigested protein. We note that proteotypic peptides included into the chimera protein standard are selected according to a few common rules, such as a higher abundance of XIC peaks, no evidence of internal and external miscleavages, no internal cysteine and methionine residues, and no aspartic or glutamic acid residue at the peptides N-terminus.^22^ We therefore hypothesized that the peak areas corresponding to an equimolar amount of proteotypic peptides released from the chimeric protein standard could cluster around some median value irrespective of their sequence. As compared to the targeted quantification by comparing the intensities of the standard and analyte peaks, MBAQ can be less affected by a biased yield of some peptide(s) because the abundance of all clustering peptides is used for calculating the median.

If so, we only have to (i) provide a sufficient number of such peptides to compute the robust median value under the given experimental conditions, (ii) select suitable peptides from those matched to the protein of interest, and (iii) check if its quantification by individual peptides is concordant. In our institute, we systematically produce large (40–270 kDa) protein chimeras comprised of 40–250 proteotypic peptides from various proteins. To test the feasibility of MBAQ, we further used CP01^9^ and CP02^4^ chimeras from our collection^22^ (Supporting Information, Expression and metabolic labeling of protein standards).

**Figure 1:**
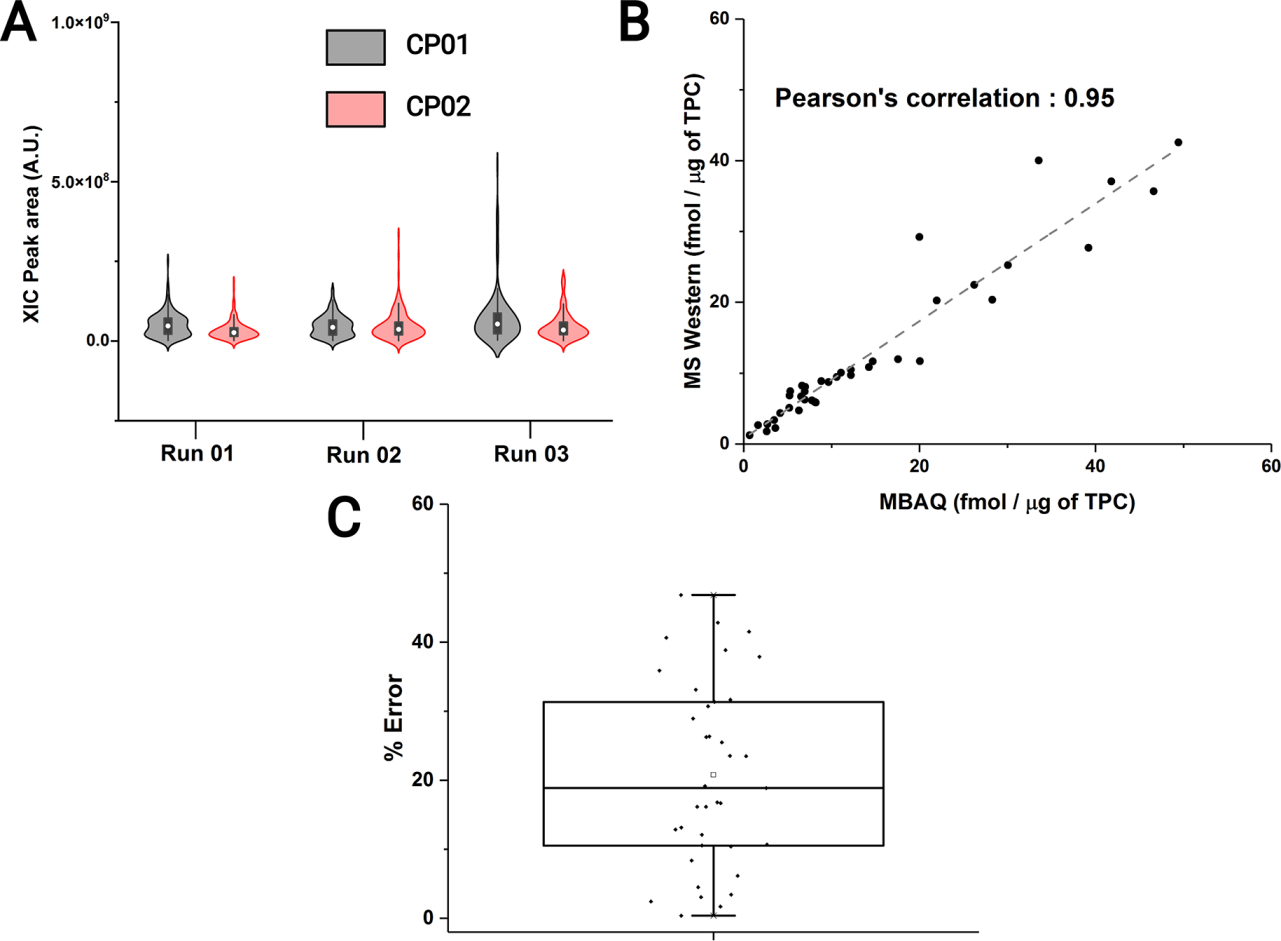
MBAQ Quantification. (A) Distribution of XIC peak areas of peptides from chimeric proteins CP01 and CP02 in three independent chromatographic runs. (B) Molar quantities of 48 metabolic enzymes from *C. elegans* quantified by MBAQ and MS Western. (C) MBAQ quantification error (in %) relative to the values determined by MS Western with each data point signifying a protein.

We first asked how the areas of the XIC peaks of the proteotypic peptides chosen from different proteins and concatenated into a chimera are distributed around the median value and how many peptides would be required to estimate it with acceptable accuracy. To this end, we digested 267 kDa chimeric protein (CP01) comprised of 250 proteotypic peptides selected from 53 *Caenorhabditis elegans* proteins.^4^ Despite an equimolar concentration of produced peptides, their peak areas differed by almost 10-fold (Figure 1A; Figure S1). However, the abundance of 48% of all peptides clustered near the median value (Figure 1A; Figure S1). In order to ascertain that clustering does not depend on some particular peptide sequences, we digested another 265 kDa chimera (CP02) harboring proteotypic peptides from 48 proteins from *Drosophila melanogaster*.^9^ We found that the peak areas of 42% of the peptides were close to the median value (Figure 1A; Figure S1). We concluded that independent of the peptide sequences, approximately one-half of the proteotypic peptides clustered around the same median while others scattered around it. However, the commonality between the peptide sequences within the clustering and nonclustering groups was not immediately obvious.

Since the “near-median” (NM) peptides were evenly distributed across the retention time range (Figure S2), we checked whether the median value could faithfully represent the molar abundance of the chimera. We expect that, in this case, possible suppression of peptide ionization by a sample matrix would be likely randomized compared to a hypothetical scenario if all peptides would be eluting together. For this purpose, we used the CP01 to quantify 48 metabolic enzymes from *C. elegans* by the MS Western protocol and, independently, using a median value computed from the abundance of all CP01 peptides. We underscore that in the MS Western workflow each enzyme was quantified using several isotopically labeled peptide standards that exactly matched sequences of the corresponding native peptides^4^ with no recourse to other peptides. In contrast, in the MBAQ workflow all peptides from the digested chimera were taken for calculating a single median value that was subsequently used for quantifying all proteins. MBAQ was concordant with MS Western, showing a Pearson’s correlation of 95% (Figure 1B) and median quantification error of 18% (Figure 1C) within 3 orders of magnitude of molar abundance difference.

In a separate experiment, we quantified 30 proteins from the commercially available UPS2 protein standard (Sigma-Aldrich, USA) using MBAQ and the median calculated from CP01 peptides. The Pearson’s correlation was 96%, and the median quantification error was less than 20% (Table S1).

We therefore concluded that if a sufficient number of equimolar prototypic peptides are detected by LC-MS/MS, their median abundance is invariant to their exact sequences and unaffected by other peptides included into the chimera. The use of median abundance as a single-point calibrant delivers good quantification accuracy that is close to the accuracy of targeted quantification relying on identical peptide standards.

Though the MBAQ workflow was accurate, use of a large isotopically labeled CP was deemed unnecessary. Effectively, we only used less than a one-half of its peptides and did not take advantage of isotopic labeling, except for validating MBAQ by independent quantification of the same proteins by MS Western. Therefore, we sought to design a generic (suitable for all proteins from all organisms) and fully unlabeled internal standard (FUGIS).

### Development of FUGIS

FUGIS was conceived as a relatively small protein chimera composed of concatenated proteotypic-like tryptic peptides that, however, share no sequence identity to any known protein. It also comprises a few reference peptides with close similarity to some common protein standard, e.g., BSA. Upon codigestion with quantified proteins, FUGIS should produce an equimolar mix of peptide standards whose median abundance would support one-point MBAQ of all of the codetected peptides from all of the proteins of interest. The exact amount of FUGIS is determined by comparison with the known amount of codigested reference protein (here, BSA) in the same LC-MS/MS experiment.

**Figure 2:**
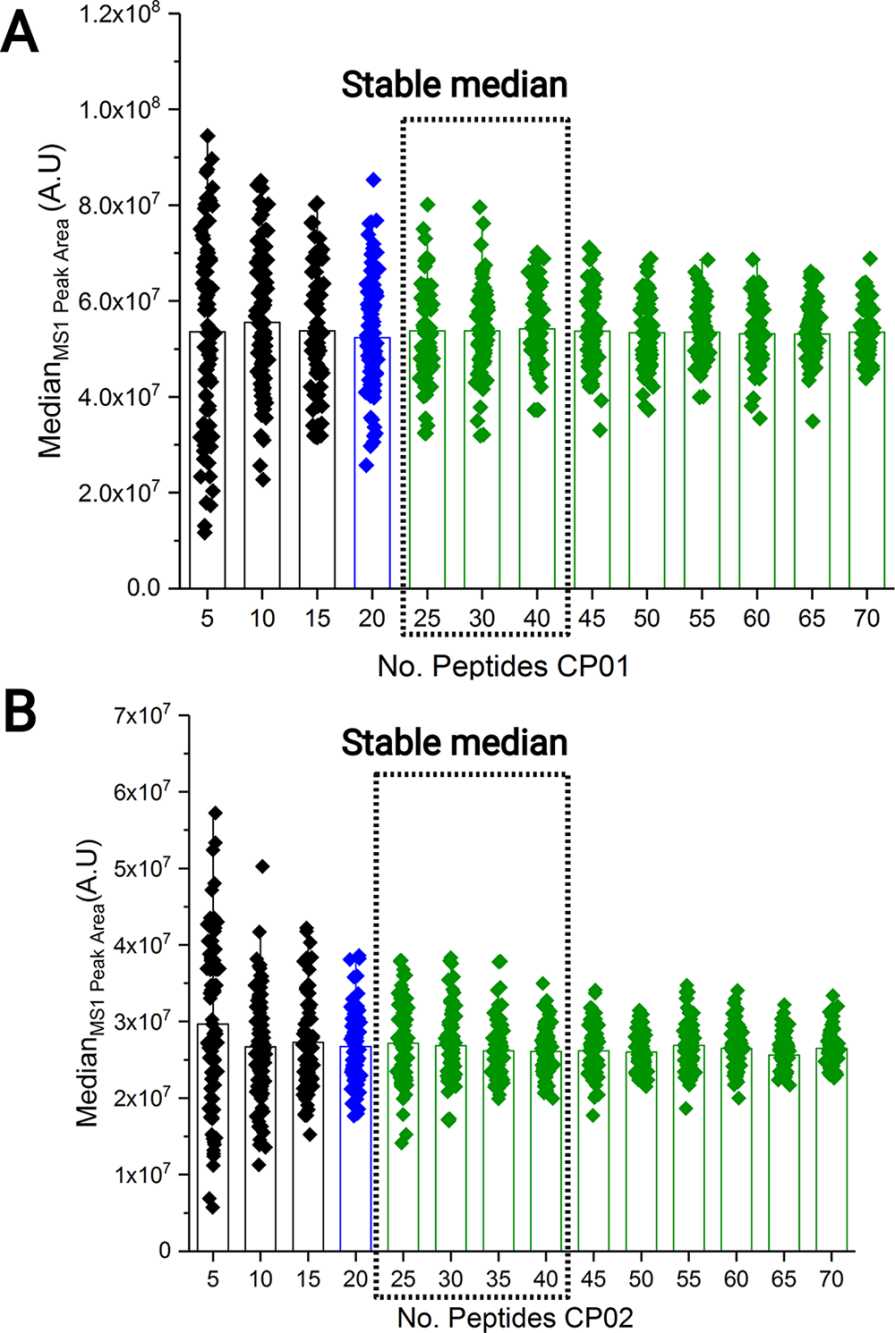
Minimum number of peptides for robust estimation of the median value. Bootstrapping of XIC peak areas of peptides from (A) CP01 and (B) CP02 over the range of 3–120 peptides in the total of 100 iterations. Filled diamonds represent median values determined by each bootstrapping iteration. Green bars represent the peptide number with stable median.

We first asked what is the minimum number of peptides required to reach a consistent median value? For this purpose, we performed a bootstrapping experiment over the abundance of tryptic peptides derived from CP01 and CP02. Median values were calculated by repetitive selection of a defined (3–120) number of peptides (Figure 2). The data collected by 100 bootstrap iterations suggested that a consistent median value can be projected by considering peak areas of as little as 5–10 peptides. However, the median spread (which depends on the “internal” peptide properties and “external” conditions of ionization) decreased with the number of peptides and reached a plateau at more than 30 peptides (Figure 2A and 2B). Also, bootstrapping revealed that irrespective of the peptides selection, the same peptides tend to cluster around the median. The abundance of 32% of 230 peptides further termed as near-median (NM) peptides was within the range of 20% of the median value. Therefore, for further work we selected 70 peptides whose peak areas were most close to the median in several technical LC-MS/MS replicates.

Next, we altered sequences of these near-median peptides in several ways such that they become different from any known sequence. Yet, we aimed to preserve the similarity of their physicochemical properties, such as net charge, hydrophobicity index, and location of polar (including C-terminal arginine or lysine) amino acid residues as compared to corresponding “source” peptides.

**Figure 3:**
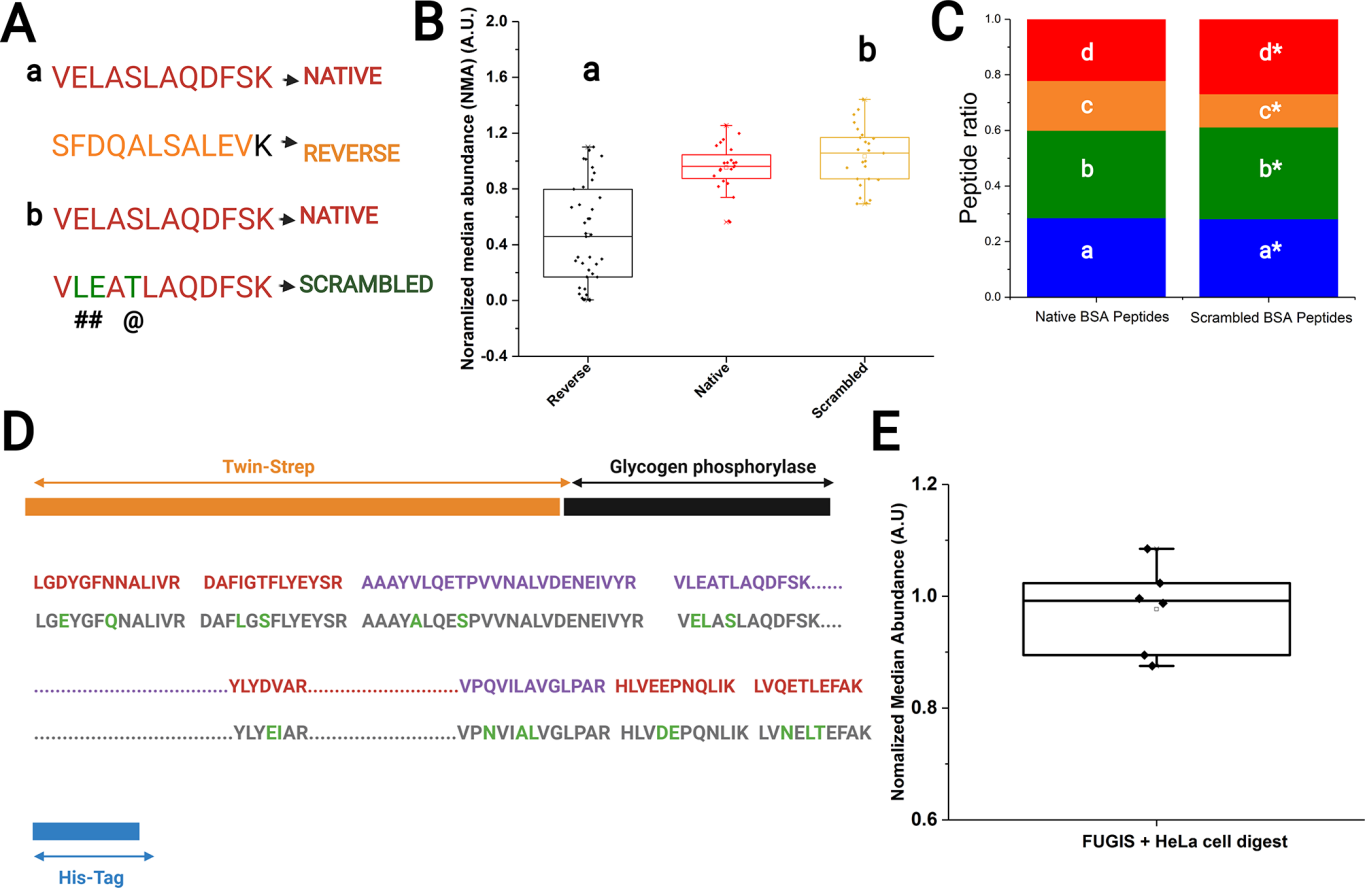
Design of FUGIS. (A) Examples of reversing (a) and scrambling (b) of peptide sequences; # indicates a swap, and @ indicates substitution of amino acid residues. (B) Normalized median abundance (NMA) of reversed, native, and scrambled peptide sequences. Each data point is a peptide. (C) Distribution of relative abundance (peptide ratios) of native and scrambled peptides. Asterisks indicate scrambled sequences: a/a*, HLVDEPQNLIK/HLVEEPNQLIK; b/b*, LGEYFGQNALIVR/LGDYGFNNALIVR; c/c*, YLYEIAR/YLYDVAR; d/d*, DAFLGSFLYEYSR/DAFIGTFLYEYSR. (D) Schematic diagram of the designed sequence of the 79 kDa FUGIS protein. Twin-Strep and His-tags are at the N- and C-termini, respectively; glycogen phosporylase peptides serve as additional reference peptides. Upper line: sequence stretches in red and in purpose are scrambled BSA and FUGIS peptides, respecitvely. Lower line: sequence stretches in gray are corresponding native peptides from BSA and source proteins; swap or substitution of amino acid residues is indicated in green. Scrambled BSA pepides are dispersed within the FUGIS sequence. Full-length sequence of FUGIS is in Figure S3A. (E) NMA of FUGIS peptides in HeLa cell background. Each data point is technically a replicate.

We first selected a set of 40 out of a total of 70 NM peptides and reversed their amino acid sequences (Figure 3A) except the C-terminal lysine or arginine and assembled them into a chimeric protein GCP01 (Table S2) that was expressed and metabolically labeled with ^13^C^15^N-Arg and ^13^C-Lys in *Escherichia coli*.^22^ Its band was excised from 1D SDS PAGE, codigested with the band of 1 pmol of BSA, and analyzed by LC-MS/MS.^22^ Similar to a previously published strategy,^37^ the peptide abundance was normalized to the abundance of the BSA peptides in the chimeric protein to check if the normalized median abundance (NMA) is close to unity (∼1.0). A unit NMA means that the median abundance truly represents the amount of the FUGIS standard, while any deviation contributes to the error in quantification.

The NMA for the reversed sequences was 0.45 (Figure 3B), which was very far from the NMA of their native counterpart of 0.97. Thus, we concluded that reversing the peptide sequences strongly biases the median and increases the spread, and therefore, it should not be used for designing a FUGIS chimera.

Next, we scrambled the peptide sequences by introducing point substitutions of amino acid residues. We allowed a maximum of two scrambling events per peptide that followed two intuitive rules. First, in each peptide only two amino acid residues were swapped (Figure 3A). Second, to create a mass shift, an amino acid residue preferably located in the middle of the peptide sequence was substituted with another amino acid having a similar side chain (e.g., Ser to Thr or vice versa) (Figure 3A). To minimize the retention time shift, aliphatic amino acids in order of increasing hydrophobicity (G < A < V < L < I) were only substituted with an amino acid having similar hydrophobicity (i.e., substitutions V by L were allowed, but G by I were not). Altogether, 20 scrambled sequences together with the corresponding 20 source “native” peptides were assembled into a chimera GCP02 (Table S3). Pairwise comparison of the peak areas of the native and scrambled sequences suggested that they differed by less than 5%. Similar to GCP01, we calculated the NMA for peptides in GCP02. Scrambled peptides behaved similar to the native sequences with a NMA of 1.02 (Figure 3B). On average, the retention time difference between the native and the scrambled peptides was 3.21 (±2.02) minutes. Therefore, these scrambled peptide sequences were selected for FUGIS.

Isotopic labeling of GCP01 and GCP02 chimeras was unavoidable since their quantification was dependent on the reference BSA peptides. We found that the reference BSA peptides scrambled in the same way behaved similar to the native peptides with the retention time shift of 1.2 (±0.5) min. Also, the relative abundance (peptide ratios)^22^ of the corresponding native and scrambled BSA peptides was very similar (Figure 3C). Therefore, metabolic labeling of a scrambled chimera was no longer required.

Taken together, we designed and produced the FUGIS chimeric protein having the molecular weight of 79.01 kDa (Figure 3D; Figure S3; Table S4), which harbored 43 scrambled near-median peptides and 5 sequences of scrambled reference peptides from BSA. Its peptides were not identical t to any known protein sequence across all organisms (Table S4).

### MBAQ Quantification Using FUGIS

To assess the feasibility and accuracy of MBAQ quantification using FUGIS, we quantified 4 proteins from 1 million HeLa cells at 2 dilutions and compared it with the quantities previously determined using MS Western.^22^ Since MBAQ quantification is based on the median abundance, we wanted to assess the accuracy of the median estimation in different matrix backgrounds. To this end, we prefractionated both dilutions of a HeLa cells lysate by 1D-SDS PAGE and excised 3 slices from each gel, which were codigested with bands of 1 pm of BSA and FUGIS. Irrespective of the protein background, the NMA calculated for FUGIS was 0.98 with less than 10% error (Figure 3E).

**Figure 4:**
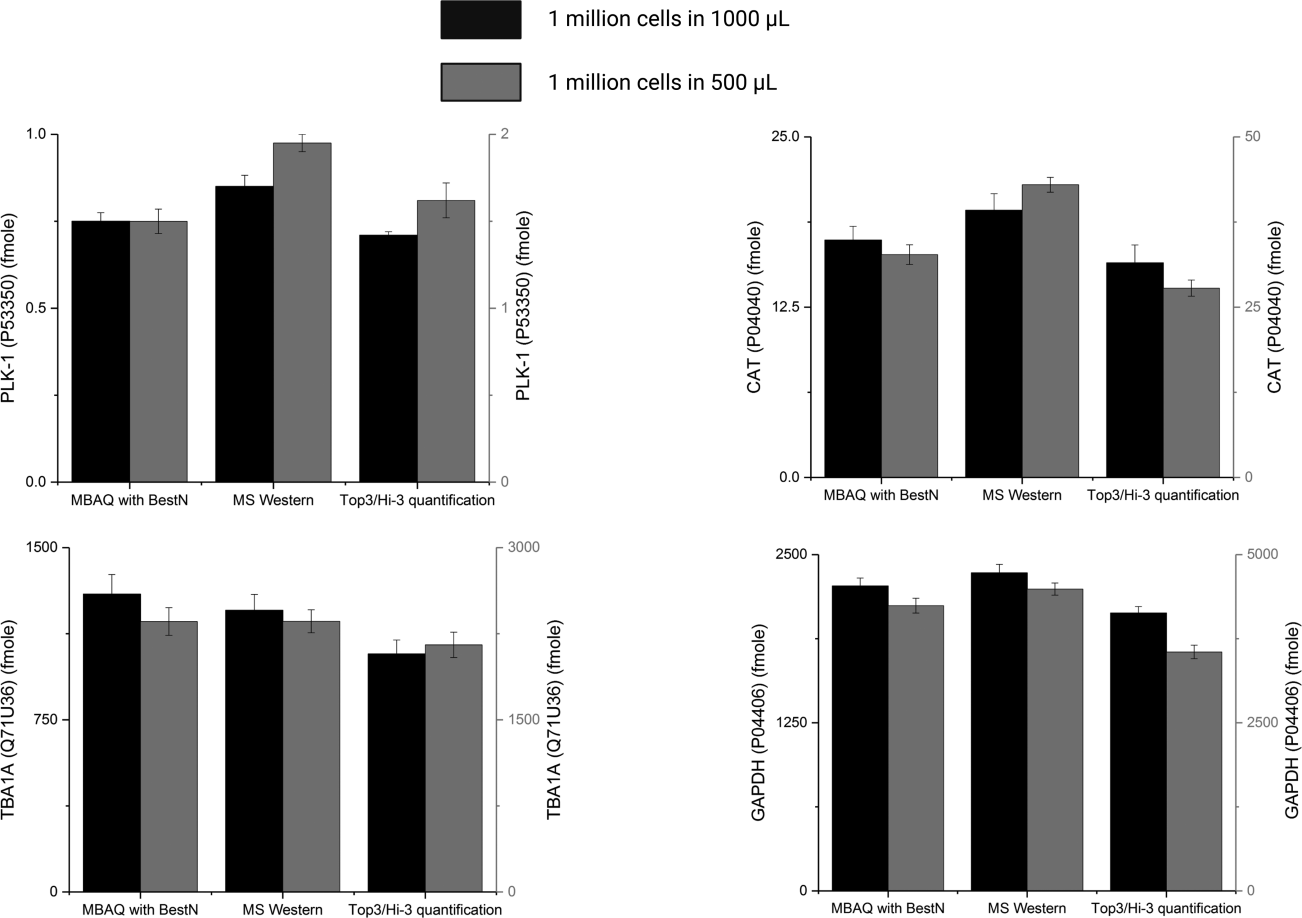
Molar quantities of proteins determined by MBAQ, MS Western, and Hi3 quantification. MBAQ vs MS Western vs Top3/Hi3 quantification of the PLK-1, CAT, G3P, and TBA1A proteins from HeLa cell lysate and from its 2-fold dilution. Error bars represent ± SEM of technical replicates.

We then proceeded to quantify the molar amounts of 4 proteins (PLK-1, TBA1A, CAT, G3P) from HeLa cells using MBAQ, MS Western,^22^ and Top3/Hi-3 quantifications^6^ (Figure 4 and Tables S5 and S6). We observed that the molar abundance determined by MBAQ was much closer to MS Western than that of Top-3/Hi-3.

**Figure 5:**
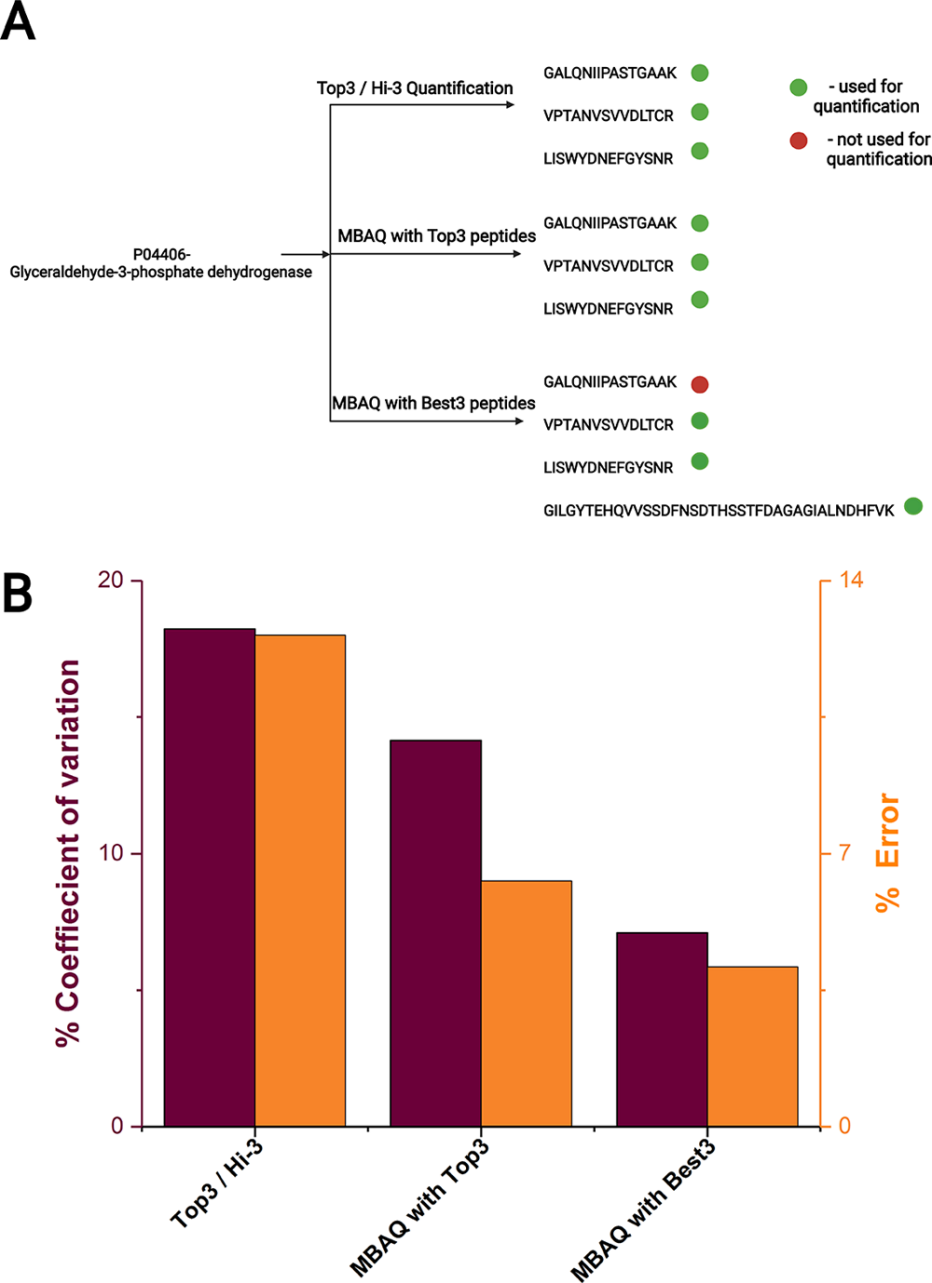
Molar quantification of human G3P protein by the MBAQ and Top3/Hi-3 methods. (A) Selection of proteotypic peptides for each method. XIC peak areas of peptides are in Figure S4. (B) Coefficient of variation (%) and % error (relative to the values determined by MS Western).

For MBAQ, in each target protein we selected peptides whose mean and median values differed by less than 15%. We termed them as “BestN” peptides–in contrast to TopN peptides that corresponded to the N most abundant peptides. To assess if BestN peptides delivered better accuracy, we looked into the quantification of one of the four proteins (glyceraldyhyde-3 phosphate dehydrogenase; G3P Human P04406) (Figure 5A; Table S5; Table S6). We estimated the concordance of its molar amount independently calculated from multiple peptides by the coefficient of variation (% CV).^9^ If calculated from the BestN peptides it was 7%, which is significantly better than Hi-3 quantification (18%) (Figure 5B; Figure S4; Table S5 and S6).

To understand why BestN peptides improved the quantification accuracy, we considered the difference between the BestN and the TopN peptide sets. For human G3P (P04406) and tubulin-1 alpha (Q71U36), the most abundant peptides were excluded from the BestN set that reduced CV down to less than 10% (Table S7). For human catalase (P04040) and serine/threonine protein kinase (P53350), the Top2 and Best2 peptides were the same (Table S7). Since the BestN peptides is a subset of the TopN peptides a minimum of two peptides was required to provide concrdant molar amounts.

Considering MS Western estimates as “true values”, we evaluated the accuracy of MBAQ quantification. MBAQ with BestN peptides delivered qunatification accuracy of 96% (Figure 5B). When used together with TopN, MBAQ performed better than Top3 quantification with an accuracy of 94% (Figure 5B).

**Figure 6:**
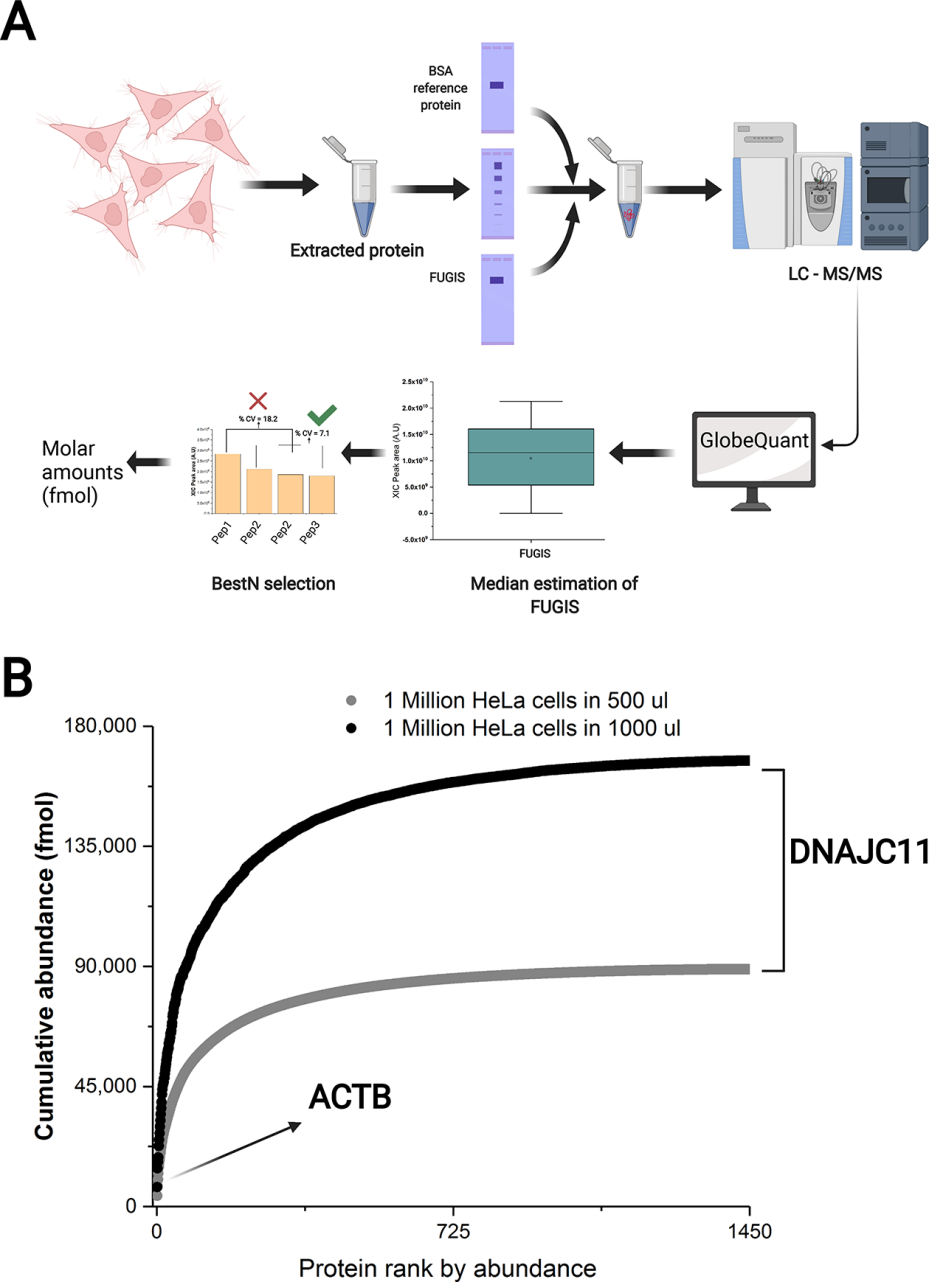
MBAQ quantification of HeLa proteome using GlobeQuant software. (A) Schematic representation of MBAQ–GlobeQuant workflow. (B) Ranked cumulative abundance of 1450 proteins from both dilutions of HeLa lysate with the least abundant protein at the right. ACTB was the most abundant protein.

The GlobeQuant software supports the MBAQ workflow (Figure 6A) by selecting the BestN peptides from analyzed proteins and using FUGIS as a single-point calibrant. We employed GlobeQuant to quantify 1450 proteins identified with two or more matching peptides in HeLa cells lysate and, independently, in its 2-fold-diluted aliquot. In each sample, proteins were quantified independently with no recourse to raw intensities of chromatographic peaks in another sample. Molar quantities of the individual proteins (Table S8) are plotted as a ranked cumulative abundance in Figure 6B. MBAQ faithfully recapitulated the anticipated 2-fold difference with an average accuracy of 92%. Protein quantities (Table S8) provide a useful resource for benchmarking of newly developed absolute quantification methods.

Finally, we checked whether absolute quantification by MBAQ and by other proteome-wide techniques such as iBAQ and Proteomic Ruler is concordant. To this end, we converted the molar abundance of the four HeLa proteins into the number of copies per cell and compared it with previous reports (Table 1). Copy numbers determined by two independent MS Western experiments were concordant and corroborated MBAQ. At the same time, MBAQ, iBAQ, and Proteomic Ruler reported discordant quantities of the same four proteins but also showed marginal concordance on the proteome-wide scale (Table 1; Figure S5). This is not surprising since both determinations by Proteomic Ruler do not correlate and are also discordant with iBAQ. Since MBAQ corroborated MS Western (Table 1), we argue that it provides a more reliable estimate of the molar abundance despite its apparent discordance to alternative methods.

**Table 1:** Number of Copies Per Cell^a^ (×10^4^) in HeLa Cells Determined by MS Western,^22^ MBAQ, Proteomics Ruler,^32^ and iBAQ^31^

	MS Western		MBAQ	Proteomics Ruler		iBAQ
protein	Kumar et al. (2018)	Raghuraman et al. (this work)	Raghuraman et al. (this work)	Hein et al. (2015)	Itzhak et al. (2016)	Nagaraj et al. (2011)
PLK-1	n.a.^b^	6.8	5.4 *(20%)*^c^	13 *(91%)*	16 *(135%)*	3.7 (46%)
CAT	100	149	113 *(24%)*	17 *(88%)*	87 *(42%)*	23 *(84%)*
TBA1A	6926	8166	8162 *(0.05%)*	n.q.^d^	n.q.	32 *(99%)*
GAPDH	n.a.	15 546	14 692 (5.4%)	1747 *(89%)*	11 848 *(24%)*	1600 *(89%)*

a Copy numbers were rounded up.

b n.a., not available.

c Error in quantification (in %) calculated relative to MS Western quantities (Raghuraman et al., this work) taken here as true values.

d n.q., not quantified.

## Conclusion and Perspectives

We argue that together with the FUGIS standard, the MBAQ workflow supported accurate absolute quantification of proteins at a proteome-wide scale. A high level of expression in *E. coli*, good solubility, and, last but not least, no interference with any known protein make FUGIS a preferred internal standard for label-free experiments aiming at the absolute but also relative quantification. Upon tryptic digestion, it produces 43 peptides in an exactly known equimolar amount covering a common range of peptide retention times. Though the current workflow involves the GeLC-MS/MS strategy, it can be easily adjusted for in-solution protocols: since FUGIS is highly expressed in *E. coli* there is no need for its further purification.

It has long been noticed that the abundance of proteins could be inferred from the abundance of the best detected (TopN) peptides, as in Hi-3 quantification.^6^ However, relying on the best ionized peptides biases its accuracy.^33,37,38^ By selecting BestN (instead of TopN) peptides, MBAQ improved the quantification consistency by disregarding peptides whose ionization capacity is based on a uniquely favorable sequence. It is also important that in MBAQ the molar abundance of peptides is referred to a recognized commercial quantitative standard.

We speculate that employing MBAQ or similar quantification might be an important step toward establishing diagnostically relevant protein values in liquid and solid biopsies. MBAQ could quantify any protein detectable with multiple (three or more) peptides in any LC-MS/MS experiment, including data-independent acquisition (DIA). MBAQ does not rely on preconceived knowledge of the protein composition or availability of MS/MS spectra libraries.

Finally, charting the proteome and metabolome composition in molar quantities will facilitate our understanding of metabolic and signaling pathways that are controlled by molar ratios between multiple enzymes and substrates and help to uncover the molecular rationale of proteotype–phenotype relationships.

## Supporting Information Available

Supplementary methods, GeLC-MS, expression and metabolic labeling of protein standards, database search and data processing; MS1 peak area of CP01 and CP02 in 3 independent chromatographic runs; retention times distribution of near-median peptides; full-length sequence of FUGIS standard, expressed FUGIS standard separated using 1D SDS PAGE, retention time distribution of peptides in the FUGIS standard; XIC peak area of the Top4 peptides of GAPDH (P04406) with rational behind the selection of the BestN peptide; comparison of the copy numbers per HeLa cell for the same proteins in Nagaraj et al. (iBAQ)[1] vs Raghuraman et al. (this paper), Hein et al. (Proteomic Ruler)[2] vs Raghuraman et al., and Nagaraj et al. (iBAQ)[1] vs Hein et al. (Proteomic Ruler)[2]; MBAQ quantification of UPS2 standard; peptide sequence of GCP01; peptide sequence of GCP02; peptide sequence of FUGIS; MBAQ vs MS Western vs Hi-3 of 1 million cells in 500 μL; MBAQ vs MS Western vs Hi-3 of 1 million cells in 1000 μL; BestN peptides of 4 HeLa proteins; GlobeQuant quantification of 1450 HeLa proteins; MS data acquisition parameters (PDF)

## Notes

Raw data have been deposited to the ProteomeXchange Consortium via the PRIDE partner repository with the data set identifier PXD026886.
